# Duration of response after DEB-TACE compared to lipiodol-TACE in HCC-naïve patients: a propensity score matching analysis

**DOI:** 10.1007/s00330-021-07905-x

**Published:** 2021-04-19

**Authors:** Irene Bargellini, Valentina Lorenzoni, Giulia Lorenzoni, Paola Scalise, Gianni Andreozzi, Elena Bozzi, Luigi Giorgi, Rosa Cervelli, Rossella Scandiffio, Orsola Perrone, Donato Vito Meccia, Antonio Boccuzzi, Francesco Daviddi, Antonio Cicorelli, Alessandro Lunardi, Laura Crocetti, Giuseppe Turchetti, Roberto Cioni

**Affiliations:** 1 Department of Interventional Radiology, Pisa University Hospital, Via Paradisa 2, 56126, Pisa, Italy; 2 Institute of Management, Scuola Superiore Sant’Anna, Piazza Martiri della Libertà 33, 56127 Pisa, Italy

**Keywords:** Carcinoma, hepatocellular, Chemoembolization, therapeutic, Propensity score, Ethiodized oil, Microspheres

## Abstract

**Objectives:**

To retrospectively compare long-term outcomes of first-line drug-eluting particle (DEB)- transarterial chemoembolization (TACE) and lipiodol-TACE, in patients with unresectable hepatocellular (HCC).

**Methods:**

We retrospectively reviewed our database to identify adult patients with treatment-naïve unresectable HCC, who underwent TACE from 2006 to 2013. Patients were excluded in the absence of complete medical records relative to first TACE, 1-month follow-up, and/or sufficient follow-up data. Periprocedural complications, duration of hospitalization, 1-month tumor response by mRECIST, time to tumor progression (TTP) and target tumor progression (TTTP), and overall survival (OS) were evaluated.

**Results:**

Out of an initial series of 656 patients, 329 patients were excluded for unavailability of sufficient baseline and/or follow-up data. The remaining 327 patients underwent either lipiodol-TACE (*n *= 160) or DEB-TACE (*n *= 167). Patients treated with lipiodol-TACE had a significantly higher tumor burden. By propensity score, patients were matched according to baseline differences (BCLC stage, uninodular or multinodular HCC, and unilobar or bilobar HCC), resulting in 101 patients in each treatment group. Lipiodol-TACE was associated with a significantly higher incidence of adverse events (*p *= 0.03), and longer hospitalization (mean, 2.5 days vs 1.9 days; *p* = 0.03), while tumor response, TTP, and OS were comparable. In patients achieving 1-month complete response (CR) of target tumor, TTTP was significantly (*p *= 0.009) longer after DEB-TACE compared to lipiodol-TACE (median, 835 vs 353 days), resulting in a lower number of re-treatments during the entire follow-up (0.75 vs 1.6, *p *= 0.01).

**Conclusion:**

Compared to lipiodol-TACE, DEB-TACE offers higher tolerability, reduced hospitalization, and more durable target tumor response after CR.

**Key Points:**

*• Compared to lipiodol-TACE, DEB-TACE is better tolerated and has reduced side effects, which translates into shorter hospitalization.*

*• When complete radiological response according to the mRECIST is obtained 1 month after the procedure, DEB-TACE offers a more durable local tumor control compared to lipiodol-TACE.*

*• In these patients, the longer duration of response after DEB-TACE translates into a lower number of re-interventions.*

**Supplementary Information:**

The online version contains supplementary material available at 10.1007/s00330-021-07905-x.

## Introduction

Hepatocellular carcinoma (HCC) represents the most frequent hepatic malignancy and the third cause of cancer-related death worldwide [1]. Prognosis mainly depends on the disease stage at the time of diagnosis, which is based not only on tumor extension but also on the patient’s clinical conditions and liver function [2].

Potentially curative treatments are available, such as liver transplant, resection, and percutaneous thermal ablation. However, because of tumor load at diagnosis and contraindications to curative therapies, the majority of patients will receive intra-arterial or systemic treatments. Transarterial chemoembolization (TACE) is the current first-choice treatment in patients with unresectable intermediate-stage HCC [3–6]. However, there is no agreement on how TACE should be performed, with high variability in terms of anti-cancer drugs and embolization modalities [7], without any clear demonstration of the superiority of a specific embolic or drug [8].

The conventional TACE technique consists of an intra-arterial administration of an emulsion of lipiodol and chemotherapeutic agent(s), followed by embolization with permanent or resorbable material (lipiodol-TACE). In the last decade, drug-eluting beads (DEBs) have been introduced in the attempt to increase intratumoral drug delivery and reduce the hepatic and systemic toxicity associated with drug delivery, and also to standardize the embolization technique by the administration of known volume of precisely calibrated non-resorbable beads. Despite controversial data [9], two prospective randomized studies demonstrated the favorable safety and tolerability profile of DEB-TACE compared to lipiodol-TACE, although no difference has been demonstrated in radiological response and clinical outcomes [10, 11]. Since 2005, in our center, DEB-TACE has progressively replaced lipiodol-TACE, becoming the almost exclusive technique since 2014, because of its favorable safety profile.

When comparing these techniques, costs should also be considered, and pharmacoeconomic data are almost absent comparing these two techniques. In a retrospective analysis of TACE costs in two different time periods, without and with the availability of DEBs, Vadot et al reported a reduction in periprocedural costs in the second time period, despite the higher cost of DEB-TACE compared to lipiodol-TACE [12]. Similarly, a drug-economic profile favorable to DEB-TACE was reported by Cuchetti et al in a meta-analysis of literature data, based on a Markov model simulation, likely due to lower hospitalization costs and adverse events [13].

The purpose of this retrospective study was to evaluate the periprocedural and long-term outcomes of DEB-TACE compared to lipiodol-TACE in unresectable naïve HCC patients treated from 2006 to 2013.

## Materials and methods

Ethical approval for this study was obtained from the institutional review board and the study was undertaken in accordance with the Declaration of Helsinki. Informed consent was obtained from available patients and waived in case of deceased or otherwise unattainable patients.

Electronical database was retrospectively analyzed to identify all patients with unresectable HCC who underwent TACE as first-line treatment at our tertiary referral university center from January 2006 to December 2013. Patients were selected for TACE after multidisciplinary tumor board discussion, and treatment was performed after obtaining written informed consent.

The choice of the TACE technique (DEB-TACE or lipiodol-TACE) was exclusively based on operators’ preferences at the time of treatment.

Eligibility criteria for the study included the following: adult patients older than 18 years, HCC not subjected to previous surgical, locoregional and/or systemic treatments, and diagnosis of HCC based on the AASLD (American Association for the Study of Liver Diseases) criteria [14, 15].

Patients were excluded from the study in case of unavailability of medical records related to hospital admission for the first TACE treatment, 1-month radiological and clinical follow-up, and/or clinical data considered sufficient for the statistical analysis.

### TACE protocol

TACE was performed according to a standard protocol, under local anesthesia, through arterial femoral access with a 5-F catheter and selective catheterization of the tumors’ feeding arteries with either 5-F catheter or 2.4-F microcatheter, depending upon the liver involvement and the vascular anatomy.

For lipiodol-TACE, a mixture of doxorubicin and iodized oil (Lipiodol; Guerbet) was injected, followed by embolization with gelatin sponge particles. For DEB-TACE, DC-Beads (Biocompatibles UK Ltd; now a Boston Scientific Company) particles of different sizes (more frequently, 100-300 μm) were loaded with 50 mg or 75 mg of doxorubicin per vial and administered intra-arterially after mixture with non-ionic contrast medium, up to a maximum of two vials; when needed, further embolization was performed with non-resorbable bland microparticles, until near-stasis.

### Follow-up

After treatment, the standard of care clinical and radiological follow-up was scheduled at 1 month and every 3 months thereafter. At the first follow-up, all patients underwent triphasic computed tomography (CT) examination, while further follow-up was performed with either CT or magnetic resonance imaging (MRI).

Retreatment was performed only on demand, after multidisciplinary tumor board discussion, taking into consideration all available options (surgery, ablation, transarterial treatments, and systemic therapy), depending upon extension of residual or recurrent viable tumor and patients’ clinical conditions.

### Data analysis

The following variables were collected: baseline demographic and clinical data (age, sex, liver function, tumor extension), TACE technique (analyzed as exposure factor), periprocedural complications, duration of hospital stay, target and overall tumor response 1 month after the first TACE procedure, radiological tumor progression, and survival.

Periprocedural complications were evaluated according to the National Cancer Institute Common Toxicity Criteria for Adverse Events (NCI-CTC AE) version 5.0. Tumor response was assessed according to the modified Response Evaluation Criteria in Solid Tumors (mRECIST) criteria [16]. Objective response (OR) was considered the sum of complete (CR) and partial response (PR).

Survival was calculated as the time from the first TACE to death or end of follow-up (July 15, 2019). Patients who were transplanted after TACE were censored at the time of liver transplantation.

### Statistical analysis

The analysis was based on an opportunistic sample for a preliminary comparison of the two TACE techniques. To allow comparison between groups undergoing different TACE techniques, the initial study population was further selected by propensity score (PS) matching according to baseline variables significantly different between the two study groups (Barcelona Clinic for Liver Cancer, BCLC, stage [2], uninodular or multinodular disease, unilobar or bilobar tumor distribution).

Standardized mean difference (SMD) was used to examine the balance of covariate distribution between groups after matching.

Data were analyzed using descriptive statistics (mean and standard deviation, sd) and compared with chi-square or Fisher exact test for categorical data and Student’s *t*-test for continuous variables. Overall survival (OS) curves were estimated by the Kaplan-Meier method and compared using the log-rank test.

To account for the non-perfect balance between groups remaining after matching, comparisons between matched groups were also performed using regression models and adjusting for PS. In detail, according to the different outcomes of interest, univariate (using TACE technique as independent variable) and multivariate (using TACE technique and PS as independent variables) regression models were obtained; linear regression analysis was used to assess hospital stay, multinomial and binomial logistic regression models were considered to evaluate complications, tumor responses, and liver transplant, while non-parametric Cox models were used to evaluate overall survival and tumor progression.

Statistical analysis was carried out with dedicated software (SAS, Cary; and Stata, StataCorp) considering a *p* value <0.05 as statistically significant.

## Results

**Fig 1:**
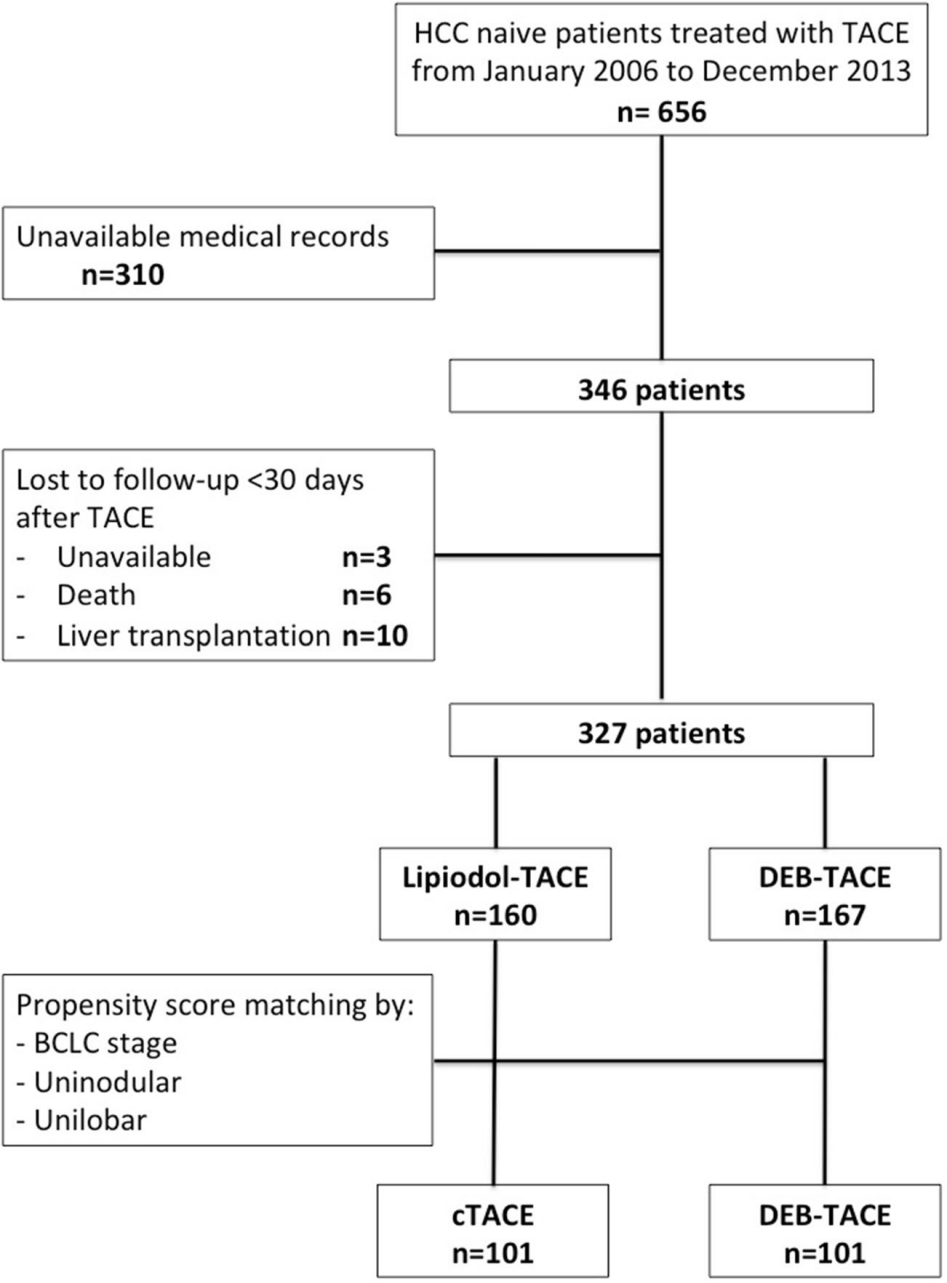
Flow chart of patients included in the study

**Fig 2:**
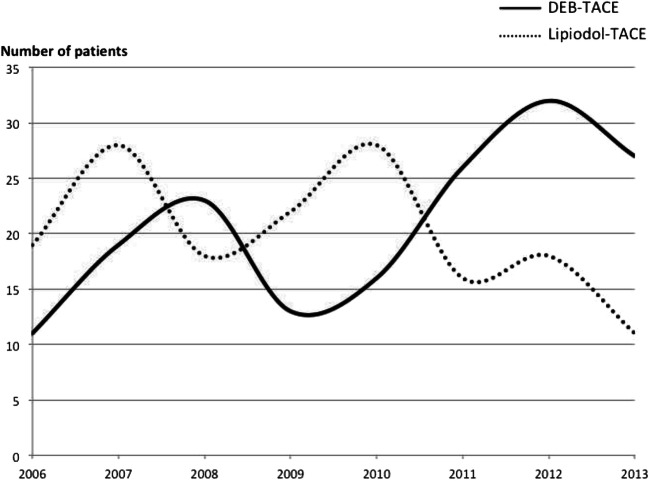
Patients’ recruitment over the years, according to the treatment performed

Out of an initial series of 656 treatment-naïve HCC patients who underwent TACE from January 2006 to December 2013, 310 patients were excluded from the study due to the unavailability of medical records relative to the first TACE treatment. Furthermore, 19 patients were lost at early (< 30 days) follow-up, because of death (*n *= 6), liver transplantation (*n *= 10), or unavailability (*n *= 3). The final study population consisted of 327 patients (M/F = 261/66; mean age 63.5 ± 10.7 years); 160 patients were treated with lipiodol-TACE and 167 patients with DEB-TACE; patients’ flow chart is summarized in Fig. 1. Figure 2 shows the number of patients recruited over the years, according to the type of TACE performed.

Clinical and demographic data are reported in Supplementary Table 1. Patients treated with lipiodol-TACE showed a significantly higher tumor burden compared to patients who underwent DEB-TACE.

### Results of the overall population

Supplementary Table 2 summarizes the periprocedural and clinical outcomes for the overall population. In the lipiodol-TACE group, a significantly higher incidence of periprocedural complications was observed, associated with longer hospitalization. Radiological tumor response 1 month after the first TACE was similar in the two groups. In case of persistent viable tumor, treatment was repeated within 3 months in 50 patients (31.2%) after lipiodol-TACE and 43 patients (25.7%) after DEB-TACE (*p*=0.27), leading to a target OR rate of 88.1% and 93.3%, respectively, as best target tumor response (*p *= 0.12). The median time to tumor progression was significantly longer after DEB-TACE. Median OS in the entire population was 28.7 months (95% CI 27.2 - 32.8) and it was significantly longer in the DEB-TACE group (*p *= 0.03).

### Patients’ characteristics after propensity score matching

Taking into account the baseline differences in the two treatment groups (Supplementary Table 1), patients were matched by BCLC stage, tumor number (uninodular or multinodular), and tumor extension (unilobar or bilobar). After matching, 101 patients in each treatment group were selected; clinical and demographic data are reported in Table 1. Clinical characteristics were homogeneous in the two groups, although some degree of imbalance (SMD > 0.1) remained for the Child-Pugh score and the diameter of the largest lesion. The matched population was represented mainly by Child-Pugh A patients, mostly with HCV-related cirrhosis; 64.3% were BCLC 0 or A patients, and 34.6% of cases were BCLC B. About half of the patients had a single lesion, with almost 80% of cases involving one single lobe.

**Table 1:** Baseline demographic and clinical features in the matched population.

Variable		Lipiodol-TACE, *n* = 101	DEB-TACE, *n* = 101	*p*	SMD
Age (years)	Mean ± sd	63.4 ± 10.2	62.6 ± 10.7	0.60	0.077
	Range	34-85	44-85		
Gender (male)	Male/female	80/21	83/18	0.59	− 0.075
Cause of cirrhosis	No	1 (1.0)	4 (4.0)	0.46	− 0.192
	HCV	52 (51.5)	59 (58.4)		− 0.140
	HBV	19 (18.8)	19 (18.8)		0
	HCV and HBV	2 (2.0)	2 (2.0)		0
	Alcohol	20 (19.8)	10 (9.9)		0.281
	Dysmetabolic	5 (4.9)	4 (4.0)		0.048
	Other*	2 (2.0)	3 (2.9)		− 0.064
Child-Pugh	A	91 (90.1)	80 (79.2)	0.09	0.306
	B	9 (8.9)	17 (16.8)		− 0.238
	C	0	0		0
Alpha-fetoprotein (μg/L)	Mean ± sd	1770 ± 9930	1067 ± 4686	0.68	0.091
	Median [25^th^-75^th^ percentile]	24.9 [8.5-174.5]	11.2 [5.5-37]		
Albumin (g/dL)	Mean ± sd	3.68 ± 0.43	3.65 ± 0.47	0.61	0.075
Total bilirubin (mg/dL)	Mean ± sd	1.13 ± 0.64	1.22 ± 0.76	0.38	− 0.127
Creatinine (mg/dL)	Mean ± sd	0.86 ± 021	0.86 ± 020	0.99	− 0.01
BCLC stage	0	8 (7.9)	8 (7.9)	1.0	0
	A	57 (56.4)	57 (56.4)		0
	B	35 (34.7)	35 (34.7)		0
	C	1 (1.0)	1 (1.0)		0
Tumor extension	Unifocal	53 (52.5)	53 (52.5)	1.0	0
	Unilobar	77 (76.2)	77 (76.2)	1.0	0
Diameter of largest lesion (mm)	Mean ± sd	34.8 ± 17.8	37.9 ± 21.2	0.26	− 0.158
	Range	12 - 110	10 - 140		
	< 30	43 (42.6)	36 (35.6)		0.142
	30-50	40 (39.6)	40 (39.6)		0
	50-70	13 (12.9)	19 (18.8)		− 0.163
	> 70	5 (4.9)	6 (5.9)		− 0.044

*Other: autoimmune (*n*=1) and cryptogenetic (*n*=4)

When not otherwise specified, data are given as numbers (and percentages)

*SMD*, standard mean difference; *NA*, not applicable

### Procedural details and periprocedural outcomes in the matched population

Table 2 summarizes the procedural details and the outcomes of the matched population. The administered doxorubicin dose was significantly higher in the DEB-TACE group. Lipiodol-TACE was associated with a higher incidence of grade 1 and 2 adverse events (*p *= 0.03), which resulted in a significantly longer hospitalization time (mean, 2.5 days versus 1.9 days; *p *= .03). Complications are described in Table 3.

**Table 2:** Procedural details and treatment outcomes in the matched population

Variable		Lipiodol-TACE, *n *= 101	DEB-TACE, *n *= 101	*p*
Dose of doxorubicin	Mean ± SD	54.5 ± 14.3	62.7 ± 24.0	0.0035
	Range	12 - 75	25 - 150	
Dose of lipiodol (mL)	Mean ± SD	14.6 ± 6.1	NA	NA
	Range	3 - 30		
Number of beads’ vials	1/2	NA	80/21	NA
Hospitalization (days)	Mean ± sd	2.5 ± 2.3	1.9 ± 1.7	0.03
Periprocedural complications	No	74 (73.3)	90 (89.1)	0.03
	Grade 1	15 (14.8)	5 (4.9)	
	Grade 2	10 (9.9)	4 (4.0)	
	Grade 3	2 (2.0)	1 (1.0)	
	Grade 4	0	1 (1.0)	
1-month target tumor response	CR	53 (52.5)	52 (51.5)	0.90
	PR	30 (29.7)	34 (33.6)	
	SD	15 (14.8)	13 (12.9)	
	PD	3 (3.0)	2 (2.0)	
1-month overall tumor response	CR	44 (43.6)	46 (45.5)	0.97
	PR	28 (27.7)	29 (28.7)	
	SD	15 (14.8)	14 (13.9)	
	PD	14 (13.9)	12 (11.9)	
Best target tumor response	CR	63 (62.4)	62 (61.3)	0.81
	PR	26 (25.7)	29 (28.7)	
	SD	11 (10.9)	8 (8.0)	
	PD	1 (1.0)	2 (2.0)	
Follow-up duration (months)	Median	30.6	33.8	0.82
	Range	2.1-123.1	1.5-105.1	
N. of treatments post-TACE	Mean ± sd	1.41 ± 1.9	1.03 ± 1.34	0.11
Post-TACE Liver transplantation (yes)		14 (13.9)	23 (22.8)	0.10

When not otherwise specified, data are given as numbers (and percentages)

*CR*, complete response; *PR*, partial response; *SD*, stable disease; *PD*, progressive disease; *NR*, not reached

**Table 3:** Complications in the overall and in the matched population

	Overall population		Matched population	
	Lipiodol-TACE, *n *= 160	DEB-TACE, *n = *167	Lipiodol-TACE, *n *= 101	DEB-TACE, *n *= 101
Abdominal pain				
Grade 1–2	4 (2.5)	0	2 (2)	0
Grade 3–4	0	0	0	0
Grade 5	0	0	0	0
Nausea/vomiting				
Grade 1–2	5 (3.1)	0	1 (1)	0
Grade 3–4	1 (0.6)	0	1 (1)	0
Grade 5	0	0	0	0
Fever				
Grade 1–2	8 (5)	5 (3)	6 (6)	4 (4)
Grade 3–4	0	1 (0.6)	0	0
Grade 5	0	0	0	0
Liver dysfunction				
Grade 1–2	21 (13.1)	3 (1.8)	13 (12.9)	3 (3)
Grade 3–4	0	0	0	0
Grade 5	0	0	0	0
Other*				
Grade 1–2	4 (2.5)*	6 (3.6)°	3 (3)^+^	2 (2)^
Grade 3–4	3 (1.9)**	6 (3.6)°°	1 (1)^++^	2 (2)^^
Grade 5	1 (0.6)***	0	0	0

Data are given as numbers (and percentages)

Other complications included:

*Cholecystitis, hyperglycemia, pleural effusion; vasovagal syndrome

**Cholecystitis, pleural effusion, respiratory failure

***Liver abscess and liver failure

°Transient renal failure, cholecystitis, pleural effusion, pancreatitis, hyperglycemia, allergy to contrast media

°°Abscess formation (*n *= 2), pancreatitis (*n *= 2), cholecystitis; groin hematoma

+Cholecystitis, vasovagal syndrome, pleural effusion

++ Pleural effusion

^ Transient renal failure, allergy to contrast media

^^ Groin hematoma, pancreatitis

### Tumor response, progression, and survival in the matched population

**Fig 3:**
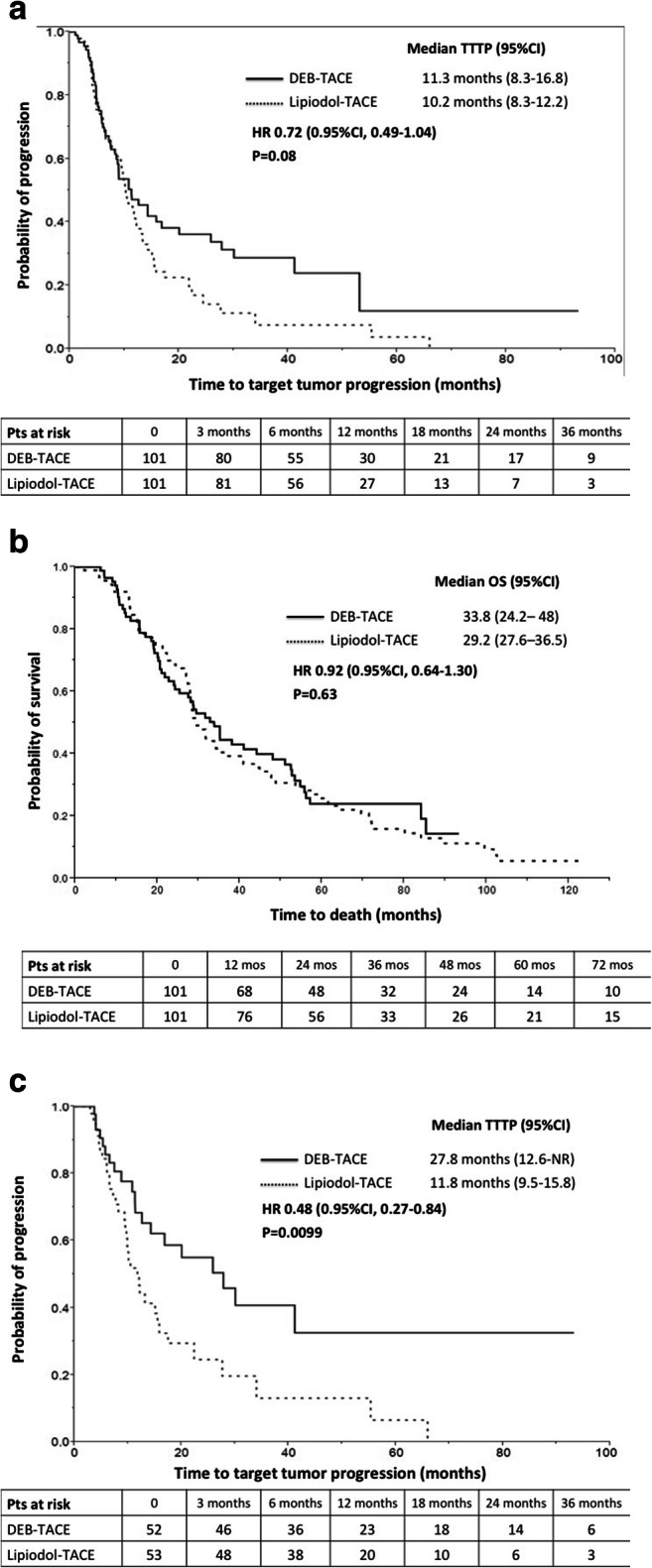
Time to target tumor progression and overall survival in the matched population**. a** Median time to target tumor progression was 10.2 months (95% CI, 8.3 – 12.2) after lipiodol-TACE and 11.3 months (95% CI, 8.3 – 16.8) after DEB-TACE (*p *= 0.08). **b** Median overall survival was 29.2 months (95% CI, 27.6 – 36.5) after lipiodol-TACE and 33.8 months (95% CI, 24.2 – 48) after DEB-TACE (*p *= 0.63). **c** In patients with complete response of the target tumor at 1 month, the median time to target tumor progression was 11.8 months (95% CI, 9.5 - 15.8) after lipiodol-TACE and 27.8 months (95% CI, 12.6-not reached) after DEB-TACE (*p *= 0.0099)

All the radiological and clinical outcomes were comparable between the two groups (Table 2). The 1-month OR rates were 82.2% and 85.2% after lipiodol-TACE and DEB-TACE groups, respectively (*p *= 0.90). The best target tumor OR rates were 88.1% for lipiodol-TACE and 90.1% for DEB-TACE (*p *= 0.81). The estimated median time to target tumor progression (TTTP) was longer after DEB-TACE, although the difference was not statistically significant (Fig. 3a). Similarly, the estimated median time to tumor progression was longer in the DEB-TACE group (16.8 months, 95% CI 12 - 24.6) compared to lipiodol-TACE (12.2 months, 95% CI 9.4 - 17.8) without a significant difference (*p *= 0.27). Patients treated with DEB-TACE received a lower number of re-treatments (1.03 vs 1.4, *p *= 0.11); liver transplantation was offered to 14 (13.9%) and 23 (22.8%) patients in the lipiodol-TACE and DEB-TACE group, respectively (Supplementary Table 3). After censoring transplanted patients, median OS was similar in the two groups, despite a trend toward longer survival after DEB-TACE (Fig. 3b). Findings were confirmed at multivariate analysis (Table 4).

**Table 4:** Results from regression models to assess the effect of treatment on selected outcome in the matched population

	DEB- vs Lipiodol-TACE (unadjusted)			DEB- vs lipiodol-TACE (adjusted for PS)		
	Coef.	*p* value	95% CI	Coef.	*p* value	95% CI
Hospitalization (days)^*^	−0.624	0.029	(−1.181;−0.066)	−0.624	0.028	(−1.179;−0.068)
Periprocedural complications						
No	*Reference value*					
Grade 1	−1.294	0.016	(−2.352;−0.237)	−1.306	0.016	(−2.369;0.242)
Grade 2	−0.302	0.069	(−2.311;0.088)	−1.116	0.069	(−2.316;0.085)
Grade 3	−0.889	0.472	(−3.309;1.531)	−0.881	0.476	(−3.302;1.540)
Grade 4	12.903	0.985	(−1356.99;1382.81)	13.211	0.987	(−1610.7;1637.12)
1-month target tumor response						
CR	*Reference value*			*Reference value*		
PR	−0.009	0.978	(−0.673;0.654)	−0.018	0.958	(−0.694;0.658)
SD	−0.113	0.791	(−0.951;0.724)	−0.118	0.784	(−0.957;0.722)
PD	−0.199	0.656	(−1.073;0.676)	−0.219	0.643	(−1.142;0.705)
1-month overall tumor response						
CR	*Reference value*			*Reference value*		
PR	0.144	0.650	(−0.478;0.767)	0.145	0.650	(−0.482;0.773)
SD	−0.124	0.771	(−0.959;0.711)	−0.124	0.771	(−0.960;0.711)
PD	−0.386	0.679	(−2.216;1.443)	−0.383	0.683	(−2.225;1.458)
Best target tumor response						
CR	*Reference value*			*Reference value*		
PR	0.125	0.699	(−0.51;0.76)	0.131	0.693	(−0.517;0.778)
SD	−0.302	0.544	(−1.278;0.673)	−0.300	0.548	(−1.278;0.678)
PD	0.709	0.567	(−1.717;3.135)	0.715	0.564	(−1.715;3.145)
Death	0.917	0.626	(0.646;1.300)	0.939	0.723	(0.662;1.331)
Target tumor progression	0.717	0.084	(0.492−1.046)	0.749	0.138	(0.512−1.097)
Tumor progression	0.807	0.270	(0.551−1.181)	0.888	0.545	(0.606−1.303)
Extrahepatic progression	0.771	0.369	(0.438;1.359)	0.842	0.553	(0.476;1.488)
Post-TACE liver transplantation	1.832	0.105	(0.882;3.807)	1.895	0.096	(0.894;4.017)

*Values reported are referred to results from simple and multiple linear regression analysis; **Values reported are referred to results from simple and multiple multinomial regression analysis; ***Values reported are referred to results from simple and multiple non-parametric Cox models

*PS*, propensity score

### Results in patients with target tumor CR 1 month after TACE

Table 5 reports the baseline characteristics and the outcomes of patients achieving target tumor CR at 1 month; the groups were not statistically significant in terms of clinical and demographic data; the DEB-TACE group received a significantly higher dose of doxorubicin. At follow-up, TTTP was significantly (*p *= 0.009) longer after DEB-TACE (median 27.8 months, 95% CI 12.6-not reached) compared to lipiodol-TACE (median 11.8 months, 95% CI 9.5-15.8) (Fig. 3c). This resulted in lower number of re-treatments during follow-up (1.6 vs 0.75, *p *= 0.01), although, ultimately, time to progression and OS did not differ significantly (*p *> 0.05).

**Table 5:** Baseline clinical data and treatment outcomes of patients with 1-month radiological complete response

Variable		Lipiodol-TACE, *n *= 53	DEB-TACE, *n *= 52	*p*
Age (years)	Mean ± sd	63.7 ± 10.1	63.3 ± 10.2	0.84
	Range	38–85	46–85	
Gender (male)	Male/female	40/13	44/8	0.24
Cause of cirrhosis	Absent	1 (1.9)	2 (3.8)	0.63
	HCV	32 (60.4)	36 (69.2)	
	HBV	8 (15.1)	7 (13.5)	
	Alcohol	8 (15.1)	3 (5.8)	
	Dysmetabolic	2 (3.8)	3 (5.8)	
	Other*	2 (3.8)	1 (1.9)	
Child-Pugh	A	49 (92.4)	44 (84.6)	0.39
	B	3 (5.7)	7 (13.5)	
	Not applicable	1 (1.9)	1 (1.9)	
BCLC stage	0	5 (9.4)	4 (7.7)	0.98
	A	35 (66)	36 (69.2)	
	B	12 (22.6)	11 (21.2)	
	C	1 (1.9)	1 (1.9)	
Tumor extension	Unifocal	30 (56.6)	32 (61.5)	0.61
	Unilobar	44 (83)	43 (82.7)	0.96
Diameter of largest lesion (mm)	Mean ± sd	32.3 ± 17.7	32.6 ± 12.8	0.91
	Range	15–110	10–67	
	< 30	27 (50.9)	21 (40.4)	
	30-49	18 (34)	24 (46.1)	
	50-70	6 (11.3)	7 (13.5)	
	> 70	2 (3.8)	0 (0)	
Dose of doxorubicin (mg)	Mean ± sd	55.4 ± 14.5	65.1 ± 22.2	0.009
	Range	12–75	30–150	
Follow-up duration (months)	Median	30.6	37.5	0.88
	Range	3.4–123.1	6.1–99.6	
Time to target tumor progression (months)	Median	11.8	27.8	0.009
	95%CI	9.5–15.8	12.6–NR	
Time to tumor progression (months)	Median	12.2	19.7	0.099
	95%CI	9.4–21.2	13.9–30.2	
Time to extrahepatic progression (months)	Median	55.1	N.R.	0.37
	95%CI	38.2–N.R.	29.4–N.R.	
N. of treatments post-TACE	Mean ± sd	1.60 ± 2.2	0.75 ± 1.20	0.016
Post-TACE Liver transplantation (yes)		6 (11.3)	14 (26.9)	0.04
Overall survival (months)	Median	29.2	35.1	0.49
	95%CI	27.8–44.2	28-53.3	

*Other: cryptogenetic (*n *= 3)

When not otherwise specified, data are given as numbers (and percentages)

*NR*, not reached

## Discussion

DEBs were developed in an attempt to increase the local efficacy of TACE and reduce systemic toxicity. Prospective randomized studies have confirmed the lower toxicity profile of DEB-TACE compared to lipiodol-TACE [10], without significant differences in radiological response and survival [10, 11].

However, real-life data are lacking regarding the duration of response in those patients for whom TACE can achieve CR, and how this result could impact on utilization of resources and long-term outcomes. Our results show that DEB-TACE can prevent local tumor recurrence for as long as 2 years after target CR, reducing the need for re-interventions.

We selected a study period between 2006 and 2013. This is related to the fact that in our hospital DEBs were introduced in 2005, with an initial unavoidable learning curve, determined also by the substantial lack of worldwide experience. After 2013, DEB-TACE has almost completely replaced lipiodol-TACE in our center, while in between the choice of performing either lipiodol-TACE or DEB-TACE was based mainly on operators’ preferences. Indeed, the analysis of the entire population shows that DEB-TACE was preferentially used in earlier stages of the disease, mainly in patients with single tumors. In fact, the use of a permanent embolic requires higher selectivity in catheterization, which was felt to be not applicable in the setting of multifocal disease, at least at the beginning of our experience.

Our data confirm the favorable toxicity profile of DEB-TACE compared to lipiodol-TACE, with lower incidence of adverse events and shorter hospitalization [17], despite the administration of higher doxorubicin doses. The number of adverse events was lower compared to previous prospective studies [11], possibly due to an underestimation of grade 1 common adverse events (such as pain and fatigue) that could have not been reported in clinical files. Nonetheless, the duration of hospitalization resulted to be significantly shorter after DEB-TACE, with a difference of approximately 1 day that may compensate the higher price of DEBs (around 800 euro) compared to lipiodol (approximately 350 euro), considering that the cost of one day of hospitalization in our facility is around 500 euro, without taking into account medications and procedures required to manage side effects. Moreover, a faster turnover in hospitalization is essential in high-volume centers. Similar results have been reported by the retrospective analysis of Vadot et al [12] and by the meta-analysis performed by Cucchetti and colleagues [13], both concluding that the lower toxicity of DEB-TACE translates into reduced hospitalization and ultimately reduced costs.

As reported in randomized controlled trials [10, 11], there were no differences in radiological response and overall survival comparing the matched treatment groups, although a trend towards longer time to tumor progression and longer survival was observed after DEB-TACE.

Longer TTP has been reported in previous retrospective studies [18, 19]. In particular, in a series of 63 consecutive patients, Ou et al showed a significantly longer TTP in the first 2 years following DEB-TACE compared to lipiodol-TACE (HR = 0.51, 95% CI: 0.29–0.88; *p *= 0.009) [18]. However, no data have been specifically reported for patients achieving CR early after treatment. In the present study, CR of the target tumor was observed in over 50% of the matched population at 1 month and was significantly more durable after DEB-TACE (over 2 years) compared to lipiodol-TACE (almost 1 year), with a reduced need for repeated treatments. This difference could be partly related not only to higher drug dose delivered selectively to the tumor but also to the possible overestimation of tumor response after lipiodol-TACE on CT, due to the artifacts caused by the Lipiodol accumulation that may mask residual viable tumor [20]. However, it has been demonstrated that homogenous lipiodol accumulation is a sign of complete necrosis [21, 22] and that there is no significant difference in the accuracy of CT in defining tumor response when comparing lipiodol-TACE versus DEB-TACE [23]. Moreover, in our series, when the lipiodol accumulation was not homogeneous early after treatment, the patient was routinely scheduled for MRI to identify residual viable tumor, and, if viable tumor was confirmed, the patient was defined as having either stable disease or PR at 1-month follow-up.

The main limitation of this study relies on its retrospective design. Several patients were excluded from the analysis since medical records could not be retrieved, follow-up was not homogeneous, and some data regarding locoregional and systemic therapies administered by clinicians in other institutions could have been missed. Another limitation is related to the time interval chosen for the study. Over the years, in fact, the knowledge on how to prepare and administer drug-eluting particles has evolved, and microparticles themselves have changed, with new devices that may improve local efficacy [24].

Despite these limitations, our analysis confirms some of the data that have been reported by large prospective randomized studies, yet adding new insights into this comparison. In fact, compared to lipiodol-TACE, DEB-TACE is better tolerated, allowing for reduced hospitalization, and is associated with more durable local tumor control after complete radiological response.

## Supplementary information

ESM 1 (DOCX 27 kb)

## Abbreviations

AASLD: American Association for the Study of Liver Diseases
BCLC: Barcelona Clinic for Liver Cancer
CR: Complete response
CT: Computed tomography examination
DEB: Drug-eluting beads
HCC: Hepatocellular carcinoma
mRECIST: Modified Response Evaluation Criteria in Solid Tumors
MRI: Magnetic resonance imaging
NR: Not reached
NCI-CTC AE: National Cancer Institute Common Toxicity Criteria for Adverse Events
OR: Objective response
OS: Overall survival
PD: Progressive disease
PR: Partial response
SD: Stable disease
sd: Standard deviation
TACE: Transarterial chemoembolization
TTTP: Time to target tumor progression

## Declarations

### Guarantor

The scientific guarantor of this publication is Roberto Cioni.

### Statistics and biometry

Two authors have significant statistical expertise.

### Study subjects or cohorts overlap

Some study subjects or cohorts have been previously reported in the following papers:

Golfieri R, Giampalma E, Renzulli M, Cioni R, Bargellini I, Bartolozzi C, Breatta AD, Gandini G, Nani R, Gasparini D, Cucchetti A, Bolondi L, Trevisani F; PRECISION ITALIA STUDY GROUP. Randomised controlled trial of doxorubicin-eluting beads vs conventional chemoembolisation for hepatocellular carcinoma. *Br J Cancer*. 2014 Jul 15;111(2):255-64.

Overlap: 4 patients

Bargellini I, Bozzi E, Campani D, Carrai P, De Simone P, Pollina L, Cioni R, Filipponi F, Bartolozzi C. Modified RECIST to assess tumor response after transarterial chemoembolization of hepatocellular carcinoma: CT-pathologic correlation in 178 liver explants. *Eur J Radiol*. 2013 May;82(5):e212-8.

Overlap: 25 patients

Bargellini I, Sacco R, Bozzi E, Bertini M, Ginanni B, Romano A, Cicorelli A, Tumino E, Federici G, Cioni R, Metrangolo S, Bertoni M, Bresci G, Parisi G, Altomare E, Capria A, Bartolozzi C.

Transarterial chemoembolization in very early and early-stage hepatocellular carcinoma patients excluded from curative treatment: a prospective cohort study. *Eur J Radiol*. 2012 Jun;81(6):1173-8.

Overlap: 38 patients

### Methodology

• retrospective

• observational

• performed at one institution
