# Supplementary material for: Duration of response after DEB-TACE compared to lipiodol-TACE in HCC-naïve patients: a propensity score matching analysis
Source: Eur Radiol. 2021 Apr 19;31(10):7512–22. doi: 10.1007/s00330-021-07905-x (PMC8452560; doi:10.1007/s00330-021-07905-x)
Supplement: Supplementary file 1 — (DOCX 27 kb) [file 330_2021_7905_MOESM1_ESM.docx]

**Supplementary Table 1:** Baseline demographic and clinical features in the overall population

| **Variable** |  | **Lipiodol-TACE**  **n=160** | **DEB-TACE**  **n=167** | **P** |
| --- | --- | --- | --- | --- |
| Age (years) | Mean ± sd | 64 ± 9.8 | 63 ± 11.5 | 0.37 |
|  | Range | 34 - 85 | 26 - 88 |  |
| Gender (male) | Male / Female | 129 / 31 | 132 /35 | 0.72 |
| Cause of cirrhosis | Absent | 2 (1.2) | 7 (4.2) | 0.16 |
|  | HCV | 86 (53.7) | 100 (59.9) |  |
|  | HBV | 29 (18.2) | 29 (17.4) |  |
|  | HCV and HBV | 2 (1.2) | 5 (3) |  |
|  | Alcohol | 29 (18.2) | 15 (9) |  |
|  | Dysmetabolic | 7 (4.4) | 7 (4.2) |  |
|  | Other* | 4 (2.5) | 4 (2.4) |  |
|  | Unknown | 1 (0.6) | 0 |  |
| Child-Pugh | A | 141 (88.2) | 132 (79) | 0.25 |
|  | B | 16 (10.0) | 27 (16.2) |  |
|  | C | 1 (0.6) | 1 (0.6) |  |
|  | Not applicable | 2 (1.2) | 7 (4.2) |  |
| Albumin (g/dL) | Mean ± sd | 3.9 ± 0.7 | 3.6 ± 0.5 | 0.08 |
| Total Bilirubin (mg/dl) | Mean ± sd | 1.07 ± 0.62 | 1.23 ± 0.74 | 0.11 |
| INR | Mean ± sd | 1.2 ± 0.17 | 1.2 ± 0.18 | 0.91 |
| AST (U/L) | Mean ± sd | 68 ± 54 | 71 ± 53 | 0.65 |
| ALT (U/L) | Mean ± sd | 65 ± 61 | 64 ± 58 | 0.93 |
| Platelets (10^3^/μL) | Mean ± sd | 112 ± 46 | 109 ± 74 | 0.74 |
| Creatinine (mg/dl) | Mean ± sd | 0.86 ± 0.24 | 0.87 ± 0.23 | 0.75 |
| Alfafetoprotein (μg/L) | Mean ± sd | 8902 ± 57857 | 1154 ± 4931 | 0.27 |
| BCLC stage | 0 | 9 (5.6) | 19 (11.4) | < .0001 |
|  | A | 69 (43.1) | 106 (63.5) |  |
|  | B | 76 (47.5) | 40 (23.9) |  |
|  | C | 5 (3.2) | 1 (0.6) |  |
|  | D | 1 (0.6) | 1 (0.6) |  |
| Tumor extension | Unifocal | 54 (33.7) | 119 (71.3) | < .0001 |
|  | Unilobar | 108 (67.5) | 143 (85.6) | < .0001 |
| Number of lesions | Mean ± sd | 3.16 ± 2.7 | 1.6 ± 1.4 | < .0001 |
| Diameter of largest lesion (mm) | Mean ± sd | 36.8 ± 17.9 | 36.4 ± 19.9 | 0.83 |
|  | Range | 12 - 110 | 10 - 140 |  |
|  | < 30 | 61 (38.1) | 70 (41.9) |  |
|  | 30-49 | 61 (38.1) | 60 (35.9) |  |
|  | 50-70 | 29 (18.2) | 28 (16.8) |  |
|  | > 70 | 9 (5.6) | 9 (5.4) |  |

* Other: autoimmune (n=1) and cryptogenetic (n=7)

When not otherwise specified, data are given as numbers (and percentages)

NA: not applicable

**Supplementary Table 2:** Procedural details and treatment outcomes in the overall population.

| **Variable** |  | **Lipiodol-TACE**  **n=160** | **DEB-TACE**  **n=167** | **P** |
| --- | --- | --- | --- | --- |
| Dose of doxorubicin (mg) | Mean ± sd | 57 ± 13.6 | 58.5 ± 20.8 | 0.42 |
|  | Range | 12 - 80 | 25 - 150 |  |
| Dose of lipiodol (ml) | Mean ± sd | 16 ± 6.2 | NA | NA |
|  | Range | 3 - 35 |  |  |
| Number of beads’ vials | 1 / 2 | NA | 139 / 28 | NA |
| Hospitalization (days) | Mean ± sd | 2.7 ± 2.5 | 2.1 ± 2.3 | 0.02 |
| Periprocedural complications | No | 113 (70.6) | 146 (87.4) | 0.001 |
|  | Grade 1 | 23 (14.4) | 8 (4.8) |  |
|  | Grade 2 | 19 (11.9) | 6 (3.6) |  |
|  | Grade 3 | 4 (2.5) | 6 (3.6) |  |
|  | Grade 4 | 0 | 1 (0.6) |  |
|  | Grade 5 | 1 (0.6) | 0 |  |
| 1-month target tumor response | CR | 76 (47.6) | 97 (58.1) | 0.23 |
|  | PR | 57 (35.6) | 49 (29.3) |  |
|  | SD | 21 (13.1) | 18 (10.8) |  |
|  | PD | 6 (3.7) | 3 (1.8) |  |
| 1-month overall tumor response | CR | 64 (40.0) | 89 (53.3) | 0.08 |
|  | PR | 51 (31.9) | 44 (26.3) |  |
|  | SD | 21 (13.1) | 19 (11.4) |  |
|  | PD | 24 (15.0) | 15 (9.0) |  |
| Best target tumor response | CR | 94 (58.7) | 117 (70.1) | 0.12 |
|  | PR | 47 (29.4) | 39 (23.3) |  |
|  | SD | 16 (10.0) | 8 (4.8) |  |
|  | PD | 3 (1.9) | 3 (1.8) |  |
| Follow-up duration (months) | Median | 28 | 35.1 | 0.04 |
|  | Range | 1-123.1 | 1.5-123.2 |  |
| Time to target tumor progression (months) | Median | 9.9 | 13.4 | 0.005 |
|  | 95%CI | 7.7 - 12 | 10.6 – 16.8 |  |
| Time to tumor progression (months) | Median | 11.1 | 19.7 | 0.004 |
|  | 95%CI | 9.3 – 15.2 | 13.9 – 28.6 |  |
| Time to extrahepatic progression (months) | Median | 35.6 | 71.6 | 0.003 |
|  | 95%CI | 24.9 – 42.1 | 49.5 – N.R. |  |
| N. of treatments post-TACE | Mean ± sd | 1.42 ± 1.79 | 1.28 ± 1.60 | 0.44 |
| Post-TACE Liver transplantation (yes) |  | 18 (11.2) | 28 (16.8) | 0.15 |
| Overall survival (months) | Median | 27.8 | 35.1 | 0.03 |
|  | 95%CI | 23.5 - 30 | 27.4 -41 |  |

When not otherwise specified data are given as numbers (and percentages)

Legend:

CR: complete response;

PR: partial response;

SD: stable disease;

PD: progressive disease;

N.R.: not reached

**Supplementary Table 3:** Liver-directed therapies after initial TACE.

| **Variable** |  | **Number of sessions** | **Lipiodol-TACE**  **n=101** | **DEB-TACE**  **n=101** |
| --- | --- | --- | --- | --- |
| N. of treatments post-TACE / per patient | Mean ± sd |  | 1.4 ± 1.87 | 1.03 ± 1. 34 |
| Liver resection |  |  | 3 | 2 |
| Liver transplantation |  |  | 14 | 23 |
| Percutanous ablation | Ethanol | 1 / 2 / 3 | 10 / 4 / 2 | 8 / 2 / 1 |
|  | Radiofrequency | 1 / 2 / 3 | 3 / 1 / 1 | 3 / 2 / 0 |
|  | Microwave | 1 | 3 | 5 |
| TACE* |  | 1 / 2 / 3 / 4 / 5 | 25 / 15 / 5 / 2 / 2 | 26 / 12 / 2 / 3 / 0 |
| Yttrium-90 radioembolization |  | 1 | 1 | 1 |
| Systemic therapy | Sorafenib |  | 21 | 13 |
|  | Regorafenib |  | 2 | 0 |
|  | Clinical trial |  | 1 | 0 |

* No cross-overs between Lipiodol-TACE and DEB-TACE occurred in the study population.
